# Genomics of Sponge-Associated *Streptomyces* spp. Closely Related to *Streptomyces albus* J1074: Insights into Marine Adaptation and Secondary Metabolite Biosynthesis Potential

**DOI:** 10.1371/journal.pone.0096719

**Published:** 2014-05-12

**Authors:** Elena Ian, Dmitry B. Malko, Olga N. Sekurova, Harald Bredholt, Christian Rückert, Marina E. Borisova, Andreas Albersmeier, Jörn Kalinowski, Mikhail S. Gelfand, Sergey B. Zotchev

**Affiliations:** 1 Department of Biotechnology, Norwegian University of Science and Technology, Trondheim, Norway; 2 N.I. Vavilov Institute of General Genetics, Department of Computational Biology, Russian Academy of Sciences, Moscow, Russia; 3 Institut fuer Genomforschung und Systembiologie, Centrum für Biotechnologie (CeBiTec), Universitaet Bielefeld, Bielefeld, Germany; 4 A.A. Kharkevich Institute for Information Transmission Problems, Russian Academy of Sciences, Moscow, Russia; 5 Faculty of Bioengineering and Bioinformatics, M.V.Lomonosov Moscow State University, Moscow, Russia; University of Strathclyde, United Kingdom

## Abstract

A total of 74 actinomycete isolates were cultivated from two marine sponges, *Geodia barretti* and *Phakellia ventilabrum* collected at the same spot at the bottom of the Trondheim fjord (Norway). Phylogenetic analyses of sponge-associated actinomycetes based on the 16S rRNA gene sequences demonstrated the presence of species belonging to the genera *Streptomyces*, *Nocardiopsis*, *Rhodococcus*, *Pseudonocardia* and *Micromonospora*. Most isolates required sea water for growth, suggesting them being adapted to the marine environment. Phylogenetic analysis of *Streptomyces* spp. revealed two isolates that originated from different sponges and had 99.7% identity in their 16S rRNA gene sequences, indicating that they represent very closely related strains. Sequencing, annotation, and analyses of the genomes of these *Streptomyces* isolates demonstrated that they are sister organisms closely related to terrestrial *Streptomyces albus* J1074. Unlike *S. albus* J1074, the two sponge streptomycetes grew and differentiated faster on the medium containing sea water. Comparative genomics revealed several genes presumably responsible for partial marine adaptation of these isolates. Genome mining targeted to secondary metabolite biosynthesis gene clusters identified several of those, which were not present in *S. albus* J1074, and likely to have been retained from a common ancestor, or acquired from other actinomycetes. Certain genes and gene clusters were shown to be differentially acquired or lost, supporting the hypothesis of divergent evolution of the two *Streptomyces* species in different sponge hosts.

## Introduction

Marine environment is a rich source of bacteria, some of which represent novel genera and species not found in terrestrial samples. Considering the hypothesis that life has originated in the ocean, it is conceivable that terrestrial bacteria represent descendants of their marine relatives, adapted to the new environment through development of specific traits, such as resistance to desiccation and ability to grow in nutrient-rich environments. However, it seems logical to assume that the reverse process, i.e. adaptation of terrestrial bacteria to the marine environment is also happening. Indeed, terrestrial bacteria that are being washed off shores or brought into the oceans by rivers would have to adapt to marine environment in order to survive and to become a part of a local microbial community. As of today, relatively little is known about the molecular mechanisms behind bacterial adaptation to marine environment, while modern genomics may provide some important clues regarding bacterial adaptation strategies [1].

Actinobacteria are a very large group of families, genera and species dwelling in many different environments, including marine sediments, flora and fauna [2]. Like their terrestrial counterparts, marine-derived species of the actinobacterial order *Actinomycetales* produce structurally diverse secondary metabolites with a wide range of biological activities, several of which are being now developed as anti-infective and anti-cancer drugs [3].

Recently, Penn and Jensen [4] compared the genomes of obligate marine actinomycetes of the genus *Salinispora* to the genomes of terrestrial actinomycetes, and identified a number of specific “marine adaptation genes” (MAG). The latter encompassed genes involved in electron transport, as well as those encoding various transporters. Loss of a particular gene encoding a mechanosensitive channel required for growth in the media with low osmotic strength has been noted for *Salinispora*. Later, Bucarey *et al* [5] have demonstrated that complementation of this lost gene restores the capacity of *Salinispora* to grow on media without sea water. Unfortunately, only a few genome sequences of marine actinomycetes are currently available, thus making studies on marine adaptation of these bacteria rather difficult.

Based on the genomics of *Salinispora* species, it has been suggested that their genetic potential to produce certain secondary metabolites is species-specific, and thus can also be relevant to the process of environmental adaptation [6]. Bioactive secondary metabolites are probably not produced by actinomycetes in the natural environment in amounts observed in artificial conditions created in a laboratory (actually targeted for overproduction of specific metabolites). It is plausible that one of the ecological functions of these compounds, many of which display profound anti-microbial activity, is not to kill competitors in fight for nutritional sources, but to modulate microbial communities. Recent findings that subinhibitory concentrations of antibiotics drastically affect transcriptional profiles of exposed bacteria supports this hypothesis [7], [8], and prompts further studies on the true roles of secondary metabolites and evolution of their biosynthetic pathways. In particular, it would be interesting to trace acquisition of new biosynthetic gene clusters by marine actinomycetes, and to study the mechanism behind their transfer, which is likely to be mediated by mobile genetic elements [9]. Although a relatively large number of actinomycetes have been isolated from marine environments, including sponges [10]–[12], only a few studies report on their genomes and biosynthetic gene clusters [13]–[15].

In this study, we isolated actinomycete bacteria from two different marine sponge species collected at the same spot at the bottom of the Trondheim fjord (Norway). Phylogenetic analysis of partial 16S rRNA gene sequences revealed isolates belonging to five actinomycete genera. Draft genome sequencing and subsequent analysis of two partially marine-adapted and closely related *Streptomyces* spp. from different sponges revealed them to be close relatives of the terrestrial species *Streptomyces albus* J1074. Comparative genomics and genome mining shed some light on the evolution of the two studied streptomycetes after presumed relocation of their *S. albus*-like ancestor from the terrestrial to the marine environment, and identified unique, compared to J1074, secondary metabolite biosynthesis gene clusters.

## Materials and Methods

### Collection of sponge samples

Sponge samples were collected from the Tautra ridge (Trondheim fjord, Norway, 63′ 36″ N and 10′ 31″ E) using MINERVA underwater remote-operated vehicle equipped with a net and a manipulator. Since the samples were collected by a national Norwegian university for research purposes, no special permit was required. The collected sponges did not represent endangered or protected species. Sponge samples of 50–3000 g were retrieved from 121 m depth and transferred to 1 l sterile plastic containers with screw cap or strong zip-lock bags filled with sterile seawater. Samples were kept at 10°C during transport (about 3 hours) and stored at 4°C until identification and processing.

### Isolation of actinomycete bacteria

Sponge pieces of ca 2 cm^3^ were cut out with a sterile scalpel on a sterile cutting board and transferred to a mortar containing 18 ml sterile seawater with 20% glycerol. For further homogenization the material was transferred to 50 ml plastic tubes with 5 g glass beads and vortexed at max. speed for 2 min. Dilutions of the processed sponge samples were plated on different agar media (IM, isolation media) and incubated at 20°C. IM4 (g/L): glycerol, 5; sodium propionate, 4; asparagine, 0.1; agar, 20 (prepared with 0.5x sea water, pH 8.0). After sterilization, 3 ml solution 2 (0.2 g/L sodium caseinate, pH 8), 0.5 M K_2_HPO_4_ and 0.5 ml solution 3 (0.005 g/L FeSO_4_x7 H_2_O, 0.01 g/L H_2_SO_4_) were added. IM7b was as described previously in [12]. IM13 (g/L): oat meal, 5; agar, 20; pH 8, prepared with 0.5x sea water. 1 ml/L trace salt solution (0.01 g/L each of FeSO_4_x7H_2_O, MnCl_2_x4H_2_O, ZnSO_4_x7H_2_O, H_2_SO_4_) and 0.5 ml/L vitamin B solution (5 mg/L each of thiamine-HCl, riboflavin, niacin, pyridoxin-HCl, inositol, Ca-pantothenate, p-aminobenzoic acid, 2.5 mg/L biotin) after sterilization. IM15 (g/L): fish flour, 2; sea weed flour, 2; agar, 20; pH 8.0, prepared with 0.5x sea water. IM16 (Gause 2 agar), g/L: glucose, 10; tryptone, 3; peptone, 5; agar, 20; pH 7.4, prepared with 0.5x sea water. IM17: 100 ml/l sediment extract (200 ml wet sediment steamed for 20 min at 100°C in 200 ml deionised water, the extract was obtained after centrifugation for 10 min at 18.000 g, sterile filtered and stored at 4°C), 20 g/L agar, pH 7.8, prepared with 0.5x natural sea water and tap water. 1 ml/L vitamin B solution (IM 13) was added after sterilization. IM18 (g/L): crab flour, 3; sea weed flour, 2; agar, 20; pH 8.0, prepared with 0.5x natural sea water. 1 ml/L vitamin B solution (IM13) and 1 ml/L trace salt solution was added after sterilization (IM13). IM 20 (g/L): shrimp flour, 3; sea weed flour, 2; agar, 20; pH 8, prepared with 0.5x natural sea water. 1 ml/L vitamin B solution (IM13) was added after sterilization. IM19: 100 ml/L sponge extract (cut sponge in pieces and cover with deionized water, steam for 20 min at 100°C, decant the extract and centrifuge for 10 min at 10,000 rpm, autoclave separately), 20 g/L agar in 0.5x sea water. 1 ml vitamin B solution (IM13) was added after sterilization.

### Molecular taxonomy and phylogenetic analyses

A phylogenetic characterisation based on molecular taxonomy was performed by sequence analysis of the 16S rRNA gene. Genomic DNA of all strains was isolated using the Qiagen DNeasy Blood and Tissue Kit. The 16S rRNA gene was amplified by PCR using the universal bacterial 16S rDNA primers F27 and R1492 [16]. Obtained PCR products were cloned into the Qiagen pDrive PCR cloning vector and sequenced using standard M13 vector primers at MWG Biotech (Germany). DNA sequences of partial 16S rRNA genes (average length 1470 bp) were compared to those available in the GenBank database (http://www.ncbi.nlm.nih.gov/Genbank/index.html) using nucleotide BLAST [17]. Sequences were aligned and a phylogenetic tree was constructed using the Molecular Evolutionary Genetics Analysis (MEGA) software version 4 [18]. The tree was computed using the neighbor-joining method and the resulting tree topology was tested by bootstrap analysis performed with 1000 replicates. The 16S rRNA gene sequence of *Bacillus subtilis* (GenBank accession number AJ276351) served as an outgroup to root the tree. GenBank accession numbers for the submitted 16S rRNA gene sequences are JX503935-JX504008.

### Genome sequencing, assembly and annotation

For each genome, two sequencing libraries were prepared: a WGS library using the Nextera DNA Sample Prep Kit and a 8 k MatePair library using the Nextera Mate Pair Sample Preparation Kit (both Illumina, San Diego, CA, U.S.A) according to the manufacturer's protocol. All libraries were sequenced in 2×250 bp paired read runs on the MiSeq platform. Reads were assembled using the Newbler assembler v2.8 (Roche Diagnostics, Mannheim, Germany). A total of 5,318,612 and 4,496,405 reads were obtained, providing 158.81x and 133.99x coverage, respectively, of the genomes of PVA 94-07 and GBA 94-10. No filtering above and beyond the standard Illumina Chastity Filtering (implemented in the base calling software) were applied with the following exception. For the 8 kb library, reads were first joined by reverse complementation of the reverse read and identification of the overlapping region between forward and reverse reads, discarding all reads with overlaps of less than 20 bp or more than two mismatches. The quality of bases in the overlapping regions was set to the highest of the two quality values in case of matching bases and to the absolute value of the difference of the quality values in case of a mismatch, retaining the base with the higher quality. The joined read was then searched for the presence of the Nextera MatePair linker sequence, with all reads being discarded where it could not be found. The read was split, linker removed, and the 5′ part was reverse complemented to simulate an “innie” read pair similar to a Sanger paired end read. In order to allow Newbler to identify those pairs and the reads of the WGS library as Sanger-like paired end reads, the FASTQ header was reformatted to resemble the pattern @ID/1 and @ID/2, respectively, with 1 indicating the “forward” and 2 the “reverse” direction.

In the case of PVA 94-07, the initial assembly consisted of 5 scaffolds containing 114 contigs. The contigs of the draft genome were analyzed for over- or underrepresentation in read coverage by means of a scatter plot to identify repeats, putative plasmids or contaminations [19]. Analysis of the scaffolds revealed two to be extrachromosomal elements (plasmids pPVA1 and pPVA2), two to make up the chromosome (core and inverted, repetitive ends) with the remaining one containing the seven copies of the ribosomal RNA (RNN) operon. For GBA 94-10, the assembly yielded 4 scaffolds containing 146 contigs. Here, only one extrachromosomal element was detected (linear plasmid pGBA1), while the remaining three scaffolds made up the chromosome, again divided into the core, the inverted repetitive ends, and finally the seven copies of the RRN operon.

The Phred/Phrap/Consed software package was used for the subsequent finishing process. After the shotgun stage, gaps between contigs were closed by editing in Consed [20], [21] for repetitive elements and low quality regions.

After in silico closure of gaps caused by low quality reads and repetitive elements, just three scaffolds (chromosome and 2 plasmids, all linear) remained, with one contig for each plasmid and 18 contigs for the chromosome of PVA 94-07. For GBA 94-10, in silico finishing reduced the number of scaffolds to two (chromosome and plasmid) with 33 contigs making up the linear chromosome. The Whole Genome Shotgun projects have been deposited at DDBJ/EMBL/GenBank under the accessions ASHE00000000 (*Streptomyces* sp. PVA 94-07) and ASHF00000000 (*Streptomyces* sp. GBA 94-10), respectively. The versions described in this paper are versions ASHE00000000.1 and ASHF00000000.1, respectively. The primary annotation was performed using the NCBI Prokaryotic Genomes Automatic Annotation Pipeline (PGAAP) [22].

### Genome mining and comparative genomics

Genomes of the PVA94-07 and GBA94-10 strains were aligned to the *Streptomyces albus* J1074 genome using MAUVE [23]. Functional gene annotation of the latter was transferred to the new strains using ad hoc Perl scripts. In addition, all proteins from the non-redundant NCBI database were used as queries in a BLAST search [17] against the new genomes, and TBLASTN hits with amino acid identity exceeding 35% and e-value less than 1E-15 were accepted. This procedure produced numerous conflicts (overlapping genes on complementary strands) due to ORF mis-annotation in closely related, GC-rich *Streptomyces* genomes. These conflicts were resolved using non-*Streptomyces* hits, so that the ORFs having homologs in non-*Streptomyces* species were accepted as correct ones. The rationale for this procedure is that spurious protein-level similarities produced at short evolutionary distance by residual, non-functional nucleotide similarities, disappear when longer evolutionary distance is considered.

Genes annotated at this stage were used to train a program for statistical gene recognition, GLIMMER [24]. The latter was applied to genomic regions that did not contain genes identified by homology at the previous stage. To analyze the gene content, orthologous gene groups were constructed. All proteins of PVA 94-07 and GBA 94-10, *Streptomyces albus* J1074 were subject to BLAST (BLASTP) search against other *Streptomyces* genomes considered as an aggregated pan-genome: *S. avermitilis* MA-4680, *S. bingchenggensis* BCW-1, *S. coelicolor* A3(2), *S. flavogriseus* ATCC 33331, *S. scabiei* 87.22, *S.* sp. SirexAA-E, *S. violaceusniger* Tu 4113, *S. cattleya* NRRL 8057, *S. clavuligerus* ATCC 27064, *S. hygroscopicus* subsp. *jinggangensis* 5008, *S. sviceus* ATCC 29083, *S. venezuelae* ATCC 10712, *S. griseus* XylebKG-1, *S. griseus* subsp. *griseus* NBRC 13350. The BLAST hits were used to construct clusters using the OrthoMCL package [25]. This package uses the bidirectional best hit criterion to identify candidate ortholog pairs. These pairs are then merged into ortholog groups using the transitive closure procedure. The default OrthoMCL parameters have been applied for the orthologous gene analysis: percentMatchCutoff = 50 (blast similarities with percent match less than 50 are ignored) and evalueExponentCutoff = −5 (blast similarities with e-value exponents larger than −5 are ignored). Finally, the similarity between initially determined orthologs is used as a threshold to identify co-orthologs (paralogs), that is, genes arising from lineage-specific duplications that are all orthologs to other genes from the group. This package yielded 13792 orthologous gene groups. Each group was characterized by a four-field pattern of occurrence in PVA94-07, GBA94-10, *S. albus* J1074 and at least one other *Streptomyces* genome. If a cluster included more than one gene from a given genome, additional genes were considered to be co-orthologs. Singletons, that is, genes not belonging to any cluster, were assumed to be species-specific genes. Secondary metabolite gene clusters were identified in the genomes using antiSMASH 2.0 [26] and manually verified.

## Results and Discussion

### Molecular taxonomy and phylogeny of actinomycetes isolated from two marine sponges

Samples from two sponges, both collected at the same location and depth of 121 m at the bottom of the Trondheim fjord and identified, based on skeletal structure and composition, as *Geodia barretti* and *Phakelia ventilabrum*, were used in this study. After treatment of samples as described in Methods, diluted suspensions of sponge material were plated on 9 different agar media and incubated for 2–6 weeks at 20°C. Colonies morphologically resembling actinomycete bacteria were transferred to the fresh plates with corresponding agar media and purified to homogenous morphology, if necessary, via successive re-plating. In total, 74 bacterial isolates preliminarily classified as actinomycetes based on morphology, were isolated: 30 from *Geodia barretti* and 44 from *Phakelia ventilabrum*. All isolates were subjected to total DNA isolation, amplification of 16S rRNA gene fragments by PCR, and DNA sequencing of the resulting PCR products.

After seven out of 30 sequences originating from the *G. barretti* isolates were removed from the data set due to redundancy, the remaining sequences were analyzed using MEGA4 software [18], yielding the phylogenetic tree presented in Figure S1 (File S1). According to this analysis, supported by verification through the Ribosomal Database Project (Release 10), eleven isolates belonged to the genus *Micromonospora*, one isolate to genus *Pseudonocardia*, three isolates were identified as *Rhodococcus* spp., one as *Nocardiopsis* sp., and seven as *Streptomyces* spp.

Similar analyses of 34 out of 44 sequences originating from the *P. ventilabrum* isolates were performed after removing ten redundant sequences (Figure S2 in File S1). 16S rRNA gene sequences from 25 isolates closely resembled those from various *Micromonospora* spp. Interestingly, two of these sequences formed a separate clade with terrestrial *Micromonospora* sp. HBUM 49404. One of the isolates was found to belong to the genus *Nocardiopsis*, two isolates to *Rhodococcus* spp., and six isolates to *Streptomyces* spp.

To determine the dependence of isolated and identified actinomycetes on sea water for growth, their cultures were replicated onto the agar media with and without sea water, and both growth and morphology were monitored over a 5-week period of incubation at 20°C. The results of this experiment presented in Table 1 show that 73% of all isolates were dependent on sea water for growth, and thus likely represent species adapted to the marine environment.

**Table 1:** Sea water dependence of sponge-associated actinobacteria isolated from *Geodia barretti* and *Phakelia ventilabrum* (SW, sea water).

Sponge	Genus	No of isolates	SW-dependent	SW-stimulated	SW-independent
*Geodia barretti*	*Nocardiopsis*	1	1	-	-
	*Micromonospora*	15	14	-	1
	*Pseudonocardia*	1	1	-	-
	*Rhodococcus*	6	6	-	-
	*Streptomyces*	7	1	1	5
*Phakelia ventilabrum*	*Nocardiopsis*	1	1	-	-
	*Micromonospora*	33	26	3	4
	*Rhodococcus*	3	1	1	1
	*Streptomyces*	7	3	3	1

Stimulation means faster growth and morphological differentiation.

### Analysis of two closely related *Streptomyces* spp. isolated from different sponges suggests a common terrestrial ancestor similar to *Streptomyces albus*

**Figure 1:**
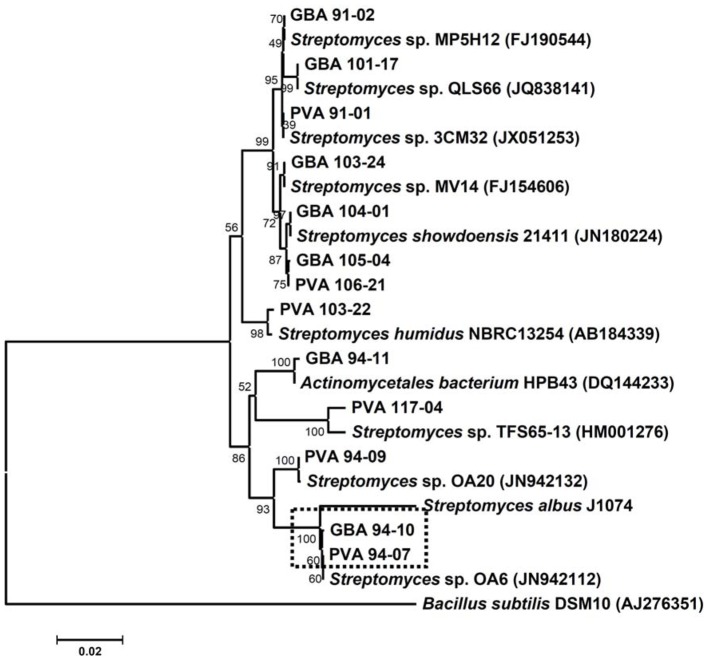
16S rRNA gene-based neighbor-joining phylogenetic tree of *Streptomyces* spp. isolated from *Phakellia ventilabrum* and *Geodia barretti* with bootstrap values (1000 replications). The nearest neighbors revealed through BLAST search of non-redundant nucleotide sequences in the public databases are presented in the tree. Nucleotide sequence accession numbers are given in brackets. The scale bar corresponds to 0.02 substitutions per nucleotide positions. Isolates chosen for further analysis are marked with a dotted box.

Considering the fact that both sponges from which actinomycete bacteria were isolated occupied the same environmental niche (depth 121 m, ca 5 m apart), it was interesting to compare the phylogeny of *Streptomyces* spp. associated with these two organisms. Phylogenetic analysis of the 16S rRNA gene sequences of *Streptomyces* spp. from two sponges suggested that several of them were closely related (Figure 1). In particular, 16S rRNA genes of *Streptomyces* sp. PVA 94-07 from *P. ventilabrum* and *Streptomyces* sp. GBA 94-10 from *G. barretti* displayed 99.7% identity. Interestingly, they also were very similar to the 16S rRNA gene from *Streptomyces* sp. OA6 isolated from Yellow Sea, China. Macro-morphologies of both strains were very similar, and on the ISP2 medium supplemented with sea water indistinguishable from that of *Streptomyces albus* J1074 grown on ISP2 without sea water. The 16S rRNA gene from the latter was partially sequenced and the data were used for building a phylogenetic tree presented in Figure 1.

**Figure 2:**
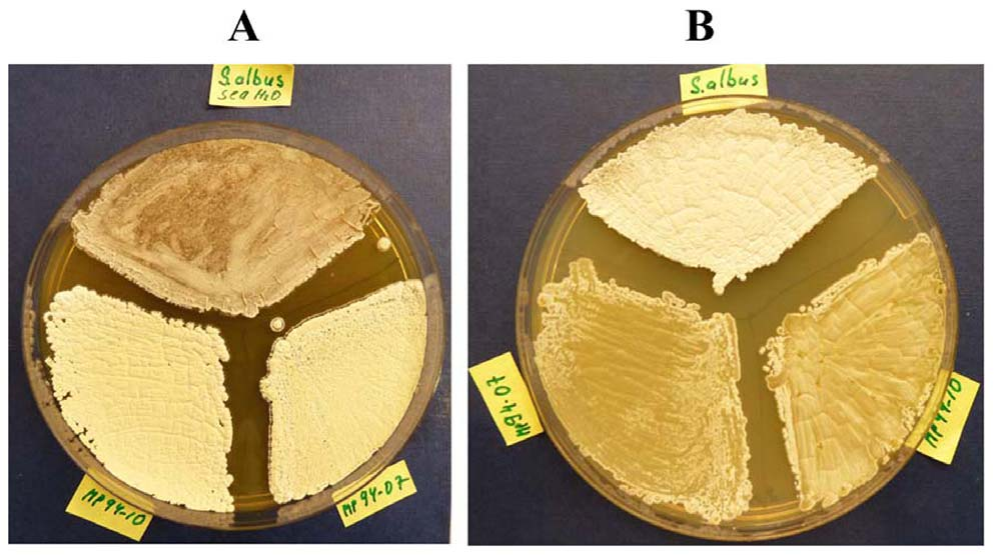
Comparison of growth and differentiation of *Streptomyces* spp. PVA 94-07 and GBA 94-10, and *S. albus* J1074 on ISP2 agar medium with (A) and without (B) sea water.

Evidently, *S. albus* J1074 clustered with both isolates mentioned above, suggesting close relatedness. To test the possible marine adaptation of the two sponge isolates, we compared their growth with that of *S. albus* J1074 on agar medium with and without sea water. The results of this experiment demonstrated that *S. albus* J1074 grows and starts to differentiate faster on the ISP2 medium without sea water, compared to PVA 94-07 and GBA 94-10. Conversely, the two latter strains grew better on the ISP2 supplemented with sea water, while *S. albus* J1074 growth and differentiation was apparently suspended on this medium after ca 48 h. The differences after 4 days of incubation are clearly visible on Figure 2. This partial adaptation to marine environment may be explained by a lower salinity of the fjord water - 18 to 32 practical salinity units (PSU), depending on the location of nearby estuary - compared to 35 PSU for Atlantic oceans water near the Norwegian coast.

Since the genome of *S. albus* J1074 has recently become available [27], we decided to compare these three bacteria at the genome level. From the evolutionary point of view, it was interesting to investigate whether the sponge isolates might have divergently evolved from a common ancestor related to *S. albus* J1074 after being transferred to the marine environment and acquired by different sponges. This was important with respect to the secondary metabolite biosynthesis potential, as recent evidence suggests that isolates having 100% 16S RNA sequence identity may have different bioactivity profiles and harbor divergent biosynthetic genes [28]. To address this question, high-quality draft genome sequences were obtained and analyzed for PVA 94-07 and GBA 94-10.

After assembly and *in silico* gap closure, a single scaffold was obtained for each replicon of PVA 94-07 (a linear chromosome and two linear plasmids, 21 kb and 77.5 kb in size) and GBA 94-10 (a linear chromosome and one linear plasmid of 198 kb in size) with one contig for each plasmid, and 18 and 33 contigs for the respective chromosomes. Gene prediction and annotation were done using the PGAAP annotation pipeline (see Materials and Methods), and the basic genomic features of both strains are summarized in Table 2.

**Table 2:** *Streptomyces* spp. PVA94-07 and GBA94-10 genome features compared to those of *S. albus* J1074.

Attribute	PVA 94-07		GBA 94-10		J1074	
	Value	% of total^a^	Value	% of total^a^	Value	% of total^a^
Size (bp)	7,104,210	100.0	7,221,787	100.0	6,841,649	100.0
G+C content (bp)	5,191,040	73.1	5,271,250	73.0	5,016,384	73.3
Coding region (bp)	6,108,726	86.0	6,150,735	85.2	5,940,480	86.8
Number of replicons	3		2		1	
Total genes	6,095	100.0	6,239	100.0	5,915	100.0
RNA genes	91	1.5	91	1.5	83	1.4
rRNA operons	7		7		7	
tRNA genes	70	1.2	70	1.1	66	1,1
Protein-coding genes	6,004	98.5	6,148	98.5	5,832	98.6
Genes with function prediction (protein)	5,128	84.1	5,261	84.3	4,653	79.8
Genes assigned to NOGs	4,708	77.2	4,852	77.8	4,422	74.8
Genes in paralog clusters	3,294	54.0	3,317	53.2	3,467	58.6
Proteins with signal peptides	527	8.6	535	8.6	517	8.7
Proteins with transmembrane helices	1,357	22.3	1,388	22.3	1,418	24.0

a The total is based on either the size of the genome in base pairs or the total number of genes in the annotated genome.

Initial genome content analysis by bidirectional best BLAST hit searched on the CDS level delivered 4,914 CDS (83.5%) for PVA 94-07 and 4,882 CDS (82.5%) for GBA 94-10 that had possible orthologs in *S. albus* J1074 [27]. Using these candidates for pair-wise genome synteny studies revealed a high degree of positional conservation between the three genomes (Figures S3–S5 in File S1). In addition, housekeeping genes in three species were compared, showing high degree of identity ranging from 98.3% to 100% (Table S1 in File S1).

The only regions without significant similarity to *S. albus* J1074 were located in the terminal inverted repetitive ends (TIRs) of 136.1 kbp (PVA94-07) and 147.1 kbp (GBA 94-10), and the regions adjacent to the 5′-TIRs. This high degree of genomes' similarity and conservation of housekeeping genes prompted us to classify the sponge isolates PVA 94-07 and GBA 94-10 as *Streptomyces albus*.

### Identification of putative marine adaptation genes by comparative genomics

**Figure 3:**
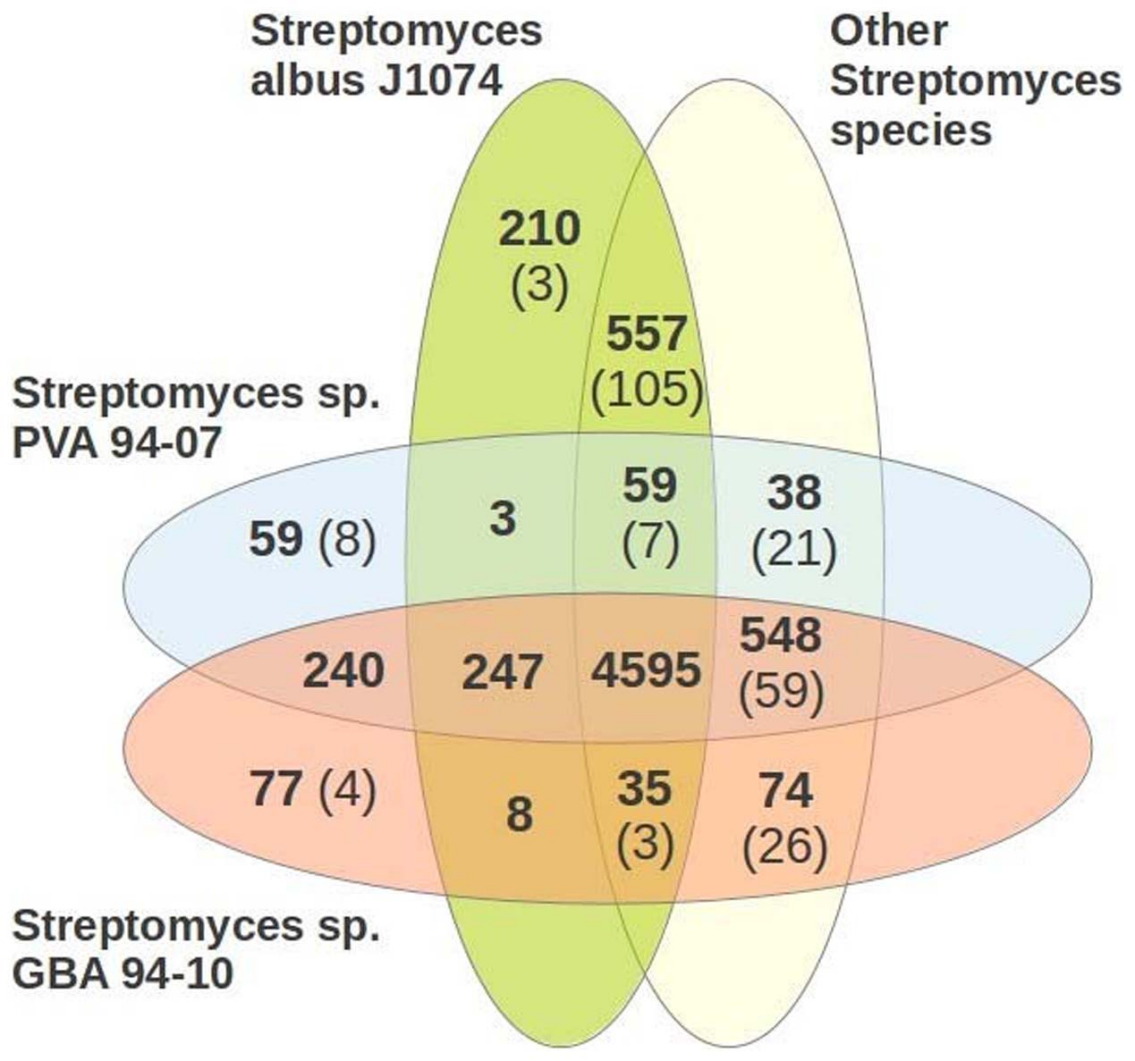
Orthologs row-based comparison of genome content of *Streptomyces* spp. PVA 94-07, GBA 94-10, *S. albus* J1074, and 11 other *Streptomyces* spp. shown as a Venn diagram. Numbers of co-orthologs are given in parentheses.

Identification of candidate genes using similarity-based and statistical approaches (see Materials and Methods) yielded 5884 and 5916 genes in PVA 94-07 and GBA 94-10, respectively. To analyze the genome content of the sponge isolates, we constructed orthologous genes groups using 14 *Streptomyces* species genomes (for details, see Materials and Methods), and the results of this analysis are shown in Figure 3 as a Venn diagram. At short evolutionary distances (within a genus) identification of orthologs based on similarity analysis implemented in OrthoMCL is largely unambiguous [25], [29], and indeed, in pairwise global genome alignments of PVA 94-07, GBA 94-10 and *S. albus* J1074 only 3.4% pairs of genes assigned to the same orthologous group occurred in non-aligned positions.

**Figure 4:**
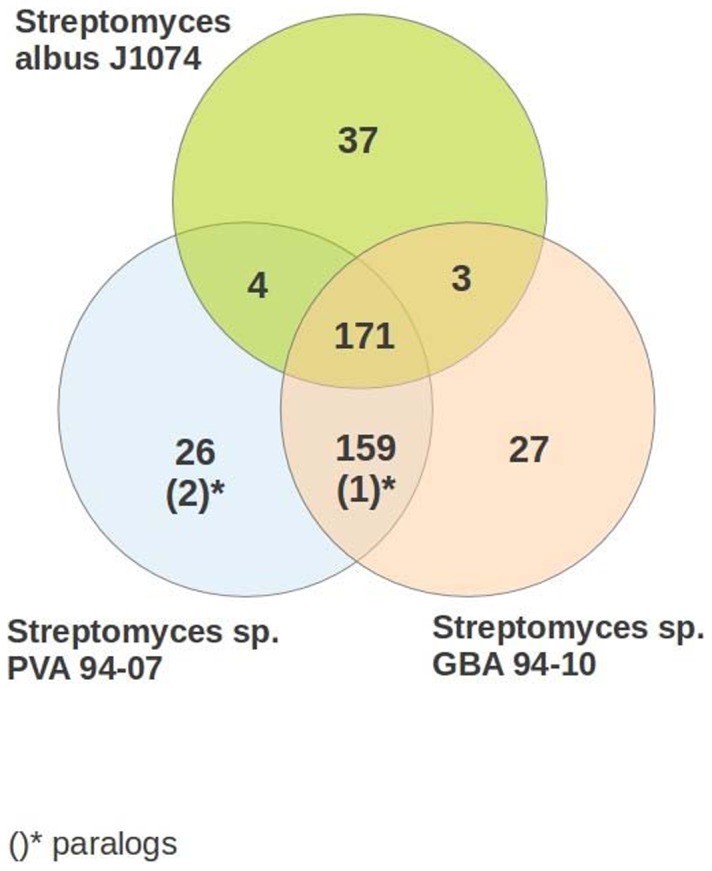
Venn diagram showing unique genes found in the genomes of PVA-94-07, GBA 94-10 and *S. albus* J1074, and some marine bacteria, but not in other *Streptomyces* investigated. Paralogs (OrthoMCL co-orthologs) are shown in parentheses.

To identify putative marine adaptation genes (MAGs) we expanded the orthologous gene set with six marine actinobacteria genomes and five incomplete marine S*treptomyces* genomes: Marine actinobacterium PHSC20C1, *Janibacter sp.* HTCC2649, *Aeromicrobium marinum* DSM 15272, *Rhodococcus erythropolis* PR4, *Salinispora arenicola* CNS-205 and *Salinispora tropica* CNB-440, *Streptomyces griseoaurantiacus* M045, *Streptomyces* sp. PP-C42, *Streptomyces sp.* W007, *Streptomyces sulphureus* L180, *Streptomyces xinghaiensis* S187. *Salinispora* species had previously been used for identification of marine adaptation genes [4] and the MAG pools of 53 and 55 genes for *S. arenicola* CNS-205 and *S. tropica* CNB-440, respectively, were taken for the analysis. The Venn diagram in Figure 4 shows genes common to PVA 94-07, GBA 94-10 and *S. albus* J1074, and absent in other soil *Streptomyces* species that were analyzed (see Materials and Methods).

In the genomes of PVA 94-07 and GBA 94-10, 215 = 159+(1)+26+(2)+27 specific genes were identified that are absent in *S. albus* J1074 and other soil streptomycetes, but present in marine actinobacteria and marine *Streptomyces* species (Table S2 in File S1). Although this set does not contain genes from the MAG pool identified by Penn and Jensen [4] for *Salinispora*, some of them might be relevant to marine adaptation, such as genes encoding putative Trk potassium uptake proteins TrkA and TrkH [30], [31].

At the same time, seven genes of the *Salinispora* MAG pool were found in the genomes of PVA 94-07, GBA 94-10 and, interestingly, *Streptomyces griseus*. These genes encode a partial NDH-1 complex: *nuoN*, *nuoM*, *nuoL*, *nuoK*, *nuoJ*, *nuoH*, and *nuoA* (Table S3 in File S1).

Proteins encoded by these genes constitute a part of a proton-pumping NADH dehydrogenase that creates a proton-motive force for generation of ATP, and have been associated with marine adaptation of *Salinispora* [4]. It seems plausible that these genes are responsible for the partial marine adaptation of PVA 94-07 and GBA 94-10 manifested by normal growth and differentiation on solid media with sea water compared to *S. albus* J1074. At the same time, both isolates contained genes encoding mechanosensitive channel [5], allowing them to proliferate on low osmotic pressure medium.

### Secondary metabolite biosynthesis genes in the sponge-associated *Streptomyces albus* isolates

The draft genomes of the PVA 94-07 and GBA 94-10 isolates, along with the genome of *S. albus* J1074 were mined for the presence of secondary metabolite biosynthesis gene clusters using antiSMASH version 2.0 [26]. According to the analyses, the PVA 94-07 genome contained 29 such clusters, while GBA 94-10 and *S. albus* J1074 genomes contained 27 and 22 such clusters, respectively. Olano et al (2014) reported identification of 27 secondary metabolite biosynthetic gene clusters in J1074 [32], although no details on how the bioinformatics analysis has been done are available yet. Several gene clusters in have been activated, leading to identification of both known and new compounds [32].

Using antiSMASH analyses data, a pairwise analysis of the identified gene clusters was performed, aimed at identification of homologous clusters and their distribution along the chromosomes of three strains. In this way, the secondary metabolite biosynthesis gene clusters common for all three strains, as well as strain-specific ones were identified. The results of this analysis are presented in Table 3.

**Table 3:** Secondary metabolite biosynthesis gene clusters identified in the genomes of PVA 94-07, GBA 94-10 and J1074 using antiSMASH 2.0.

PVA 94-07	GBA 94-10	*S. albus* J1074	*tnp*	Putative product
Gene cluster type	Gene cluster type	Gene cluster type		
Nrps1*	Nrps1*		+	
		*T1pks-Nrps1*	+	
T1pks-butyrolactone*	T1pks-butyrolactone*		+	
Terpene1	Terpene1			
Nrps2	-			
Nrps-T3pks-t1pks	Nrps-T3pks-T1pks		+	kijanimycin-related
***Nrps-T1pks***	***Nrps-T1pks***	***Nrps-T1pks***		
***Terpene2***	***Terpene2***	***Terpene2***		hopanoids
***Bacteriocin1***	***Bacteriocin1***	***Bacteriocin1***		
T1pks1	T1pks1		+	
		***Bacteriocin2***		
		***Nrps3***		
Bacteriocin2	Bacteriocin2			
***Siderophore1***	***Siderophore1***	***Siderophore1***		
***Terpene3***	***Terpene3***	***Terpene3***		
Lantipeptide	-		+	
***Terpene4***	***Terpene4***	***Terpene4***		
***Thiopeptide-lantipeptide***	***Thiopeptide-lantipeptide***	***Thiopeptide-lantipeptide***		
		***Bacteriocin3***	+	
		***Nrps4***		
Nrps5	Nrps5		+	lipoglycopeptide
Nrps6	Nrps6		+	
***Nrps7***	***Nrps7***	***Nrps7***		
***Nrps8***	***Nrps8***	***Nrps8***		
***Siderophore2***	***Siderophore2***	***Siderophore2***		deferoxamine
Nrps9	Nrps9			
***Ectoine***	***Ectoine***	***Ectoine***		ectoine
T2pks	T2pks		+	enterocin
		***Nrps10***		indigoidin
***Terpene5***	***Terpene5***	***Terpene5***	+	
***T3pks***	***T3pks***	***T3pks***		
***T1pks2***	***T1pks2***	***T1pks2***		candicidin
***Nrps-t1pks-lantipeptide***	***Nrps-t1pks-lantipeptide***	***Nrps-t1pks-lantipeptide***		antimycin
***T1pks3***	***T1pks3***	***T1pks3***	+	
T1pks-butyrolactone*	T1pks-butyrolactone*		+	
Nrps1*	Nrps1*		+	

Distribution 5′ to 3′ end. Nrps – non-ribosomal peptide synthetase; pks – polyketide synthase (T1-3 =  type 1–3); tnp – transposase gene or remnants thereof.

*Duplicated inverted clusters located within chromosomal TIRs. Clusters in J1074 and their homologues in PVA 94-07 and GBA 94-10 are indicated in bold Italic.

Interestingly, gene clusters initially denoted by antiSMASH as Nrps1 and T1pks-butyrolactone near the 5′ ends of the PVA 94-07 and GBA 94-10 chromosomes, and butyrolactone-T1pks-Nrps near the 3′ ends, are both contained within ca 135 kb of chromosomal terminal inverted repeats (TIRs), and represent duplicated inverted versions. Detailed analysis of these clusters suggested that identification of the 5′-TIR version as separate NRPS and type I PKS-butyrolactone biosynthesis gene clusters is likely to be correct for both TIRs.

We have also analyzed the TIRs of the *S. albus* J1074 chromosome, which appear to be much shorter (ca 29 kb) compared to the TIRs of the sponge streptomycetes (136 and 147 kb), but also contain parts of gene clusters for biosynthesis of secondary metabolites. The 5′ TIR encompasses part of a gene cluster annotated by antiSMASH as type I PKS-NRPS (T1pks-Nrps1, Table 3), including gene xnr_0023 coding for a truncated PKS protein with the start codon at position 29880 on the complementary strand. The 3′ TIR contains a part of a type I PKS cluster (T1psk3), which is also present in the PVA 94-07 and GBA 94-10 genomes, and starts within the 3′ portion of gene xnr_5917 encoding a complete PKS protein. This TIR encodes a portion of an inverted copy of the type I PKS-NRPS cluster identified with the 5′ TIR. Evidently, the 3′ portion of the T1pks-Nrps1 cluster after xnr_0023 and the 5′ portion of the T1pks3 cluster differ drastically in their gene content, as the former contains a gene encoding a NRPS protein, while the latter encode type I PKSs only. It seems plausible that this arrangement reflects the process of “morphing” of different biosynthetic gene clusters that may yield hybrid clusters specifying biosynthesis of new compounds.

Apparently, although *S. albus* J1074 and the two marine isolates have a common ancestor, the ability of these strains to synthesize secondary metabolites has drastically changed after separation of the lineages. Moreover, the analysis suggests that GBA 94-10 and PVA 94-07 evolved divergently after a certain time point, probably after acquisition of a common ancestor by two different sponges. Indeed, the GBA 94-10 isolate genome shares sixteen gene clusters with the *S. albus* J1074 genome, while, compared to the latter, eight are missing and ten unique are present. In this respect, the PVA 94-07 isolate differs from GBA 94-10 in that it has twelve unique gene clusters relative to *S. albus* J1074. Between themselves, GBA 94-10 and PVA 94-07 share ten identical secondary metabolite gene clusters (disregarding duplicated versions of two clusters within one of TIRs) not present in *S. albus* J1074 (Table 3). Since the arrangement of the common gene clusters on three chromosomes is similar, it seems logical to assume that these clusters are retained from a common ancestor. Notably, seven out of ten such clusters contain transposase genes that might have been involved in the process of gene cluster transfer from mobile genetic elements into the chromosomes. Detailed analysis of the gene clusters allowed us to more precisely predict types of compounds specified by some of them (Table 3), while for others such predictions remained dubious.

### Enterocin biosynthetic gene cluster in the genomes of sponge-associated *S. albus* resembles a composite transposon

Among the common ten gene clusters shared by PVA94-07 and GBA94-10, and not present in *S. albus* J1074, one could with certainty be associated with a known compound, aromatic polyketide enterocin [33]. Interestingly, the enterocin biosynthesis gene cluster was first identified in a marine bacterium *Streptomyces maritimus* isolated near Hawaii [34]. The enterocin (*enc*) gene clusters from PVA 94-07 and GBA 94-10, considered from the start codon of *encE* to the stop codon of *encT*, shared 99% identity at the nucleotide level with each other, and 95% identity with the *enc* cluster from *S. maritimus*. The most divergent DNA region, where the *enc* clusters from sponge isolates and *S. maritimus* were only 75% similar (with 8 gaps), was the putative regulatory region upstream of transcriptional regulator gene *encF*. It is plausible that this reflects evolution of the regulatory element controlling the *enc* expression in a new host.

**Figure 5:**
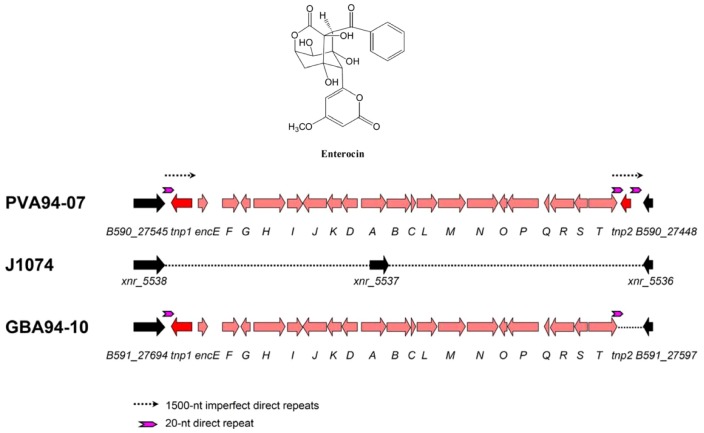
Enterocin biosynthesis gene clusters identified in the genomes of *Streptomyces* spp. PVA 94-07 and GBA 94-10. Genomic region of *S. albus* J1074 where the cluster has been inserted is shown in the middle.

Remarkably, the *enc* cluster in PVA94-07 was flanked by two imperfect direct repeats of *ca* 1500 nt, with a complete gene *tnp1* encoding an IS110-type transposase within the left repeat, and a truncated version of a highly similar transposase gene, *tnp2*, within the right repeat (Figure 5). The truncated *tnp2* transposase gene was also found to be flanked by 20-nt perfect direct repeats. Proximal to the transposase genes, and outside of the *enc* cluster, the genes (>93% identity to *S. albus* J1074 homologues at the nucleotide level) encoding homologues of a hypothetical protein (XNR_5538) and a putative sulfurtransferase (XNR_5536) of *S. albus* J1074 were located. It seems likely that the *enc* cluster has integrated between the XNR_5536 and XNR_5538 genes in the genome of a *S. albus*-like ancestor, resulting in the deletion of the gene encoding a putative 3-oxoacyl-ACP-reductase gene XNR_5537. In order to ascertain that the *enc* cluster is not present in the *S. albus* J1074 genome, a 0.8 kb fragment of the *encP* gene was amplified by PCR from the genome of PVA 94-07, DIG-labeled, and used as a probe in the Southern blot analysis of the *S. albus* J1074, PVA 94-07 and GBA 94-10 genomic DNAs. The results (not shown) clearly demonstrated the presence of the *enc* cluster in the sponge isolates and its absence in *S. albus* J1074. Also, no PCR product could be obtained from *S. albus* J1074 genomic DNA using *encP*-specific primers, while efficient amplification of the *encP* gene fragment was shown for both PVA 94-07 and GBA 94-10.

In the GBA 94-10 isolate, the organization of the *enc* cluster was found to be highly similar, but only one transposase gene, *tnp1*, was identified at the left flank of the cluster and no long direct repeats could be found in the vicinity. At the same time, the complete *enc* cluster in this isolate was flanked by 20-nt perfect direct repeats, which were also found in exactly the same position in the *enc* cluster of PVA 94-07, but also surrounding the *tnp2* gene in the genome of the latter strain.

Considering the data above, it seems plausible that the *enc* gene cluster was acquired by the common ancestor of PVA 94-07 and GBA 94-10 after it had been transferred from the terrestrial to the marine environment. The structure of the DNA region encompassing the *enc* cluster and two transposase genes in PVA 94-07 resembles a composite transposon, and suggests that a transposition event, possibly involving a mobile genetic element such as linear plasmids identified in both sponge isolates, might have been involved in the cluster acquisition. The identical genes surrounding the site of cluster insertion in PVA94-10 and GBA94-10 can be mapped to the *S. albus* J1074 genome, implying that the *enc* cluster had been transferred prior to the event that spatially separated these isolates (i.e. via acquisition by sponges). Since most of the secondary metabolite biosynthesis gene clusters shared among PVA94-07 and GBA94-10 but absent in *S. albus* J1074 are physically associated with transposase genes (Table 3), it is logical to assume that transposon-like elements play an important role in transfer of such clusters between genomes. Interestingly, the 198 kb linear plasmid pGBA1 in GBA 94-10 harbors genes (or remnants thereof) for 23 transposases, while no transposase genes could be identified on two linear plasmids in PVA 94-07. It would be interesting to investigate whether any of the secondary metabolite gene clusters in the GBA 94-10 chromosome could be mobilized by pGBA1 and transferred to other *Streptomyces* species. This hypothesis is plausible, since giant linear plasmids in *Streptomyces* have been shown to carry numerous secondary metabolite biosynthesis gene clusters, can recombine with TIRs of linear chromosome, as well as integrate into it forming distinct genomic islands [35], [36].

### Biological activity of marine *S. albus* isolates

From the results of genome mining, it was obvious that marine isolates of *S. albus* PVA 94-07 and GBA 94-10 differ significantly from *S. albus* J1074 in terms of secondary metabolite biosynthetic capacity. In order to test biological activity of the isolates, including J1074, they were incubated in 4 different production media, and DMSO extracts of the cultures tested for bioactivity against *Candida albicans*, *Micrococcus luteus* and *Enterococcus faecium* as previously described in Bredholdt et al. [12]. Moderate anti-fungal activity against *C. albicans* was detected for all media tested, presumably due to the production of candicidin or antimycin [32], while no anti-bacterial activity was shown for any of the extracts. These results may indicate that most of the secondary metabolite biosynthesis gene clusters remain “silent” in *S. albus*, or may stem from assay conditions. We are currently using several approaches to activate these clusters in an attempt to reveal true biosynthetic potential of the sponge-associated *S. albus* strains.

### On the divergent evolution of *S. albus*

Bacteria are the most versatile living organisms on Earth, capable of occupying diverse environmental niches, such as glaciers, hot springs, marine and desert habitats etc. Their ability to adapt to a particular environment is unprecedented, owing mainly to their fast mutation rate, flexible regulatory networks linked to environmental sensors, and efficient gene loss and acquisition (e.g. via horizontal gene transfer). Actinomycete bacteria, best known for their capacity to produce various bioactive metabolites, dwell in very different environments, and the mechanisms by which they adapt and evolve are poorly understood.

The comprehensive genome analysis and mining of two sponge-associated *Streptomyces* species revealed that they have evolved from a common ancestor resembling the terrestrial *S. albus* J1074. The latter strain is a derivative of *S. albus* G [37] deficient in production of *Sal*I endonuclease, amenable to genetic manipulation, and frequently used for heterologous expression of antibiotic biosynthesis gene clusters. Notably, although *Sal*I restriction-modification system-encoding genes could be readily identified in the J1074 genome (albeit inactivated by insertion of an IS element), they were completely absent in the genomes of sponge isolates. This fact may indicate that the *Sal*I system has been acquired specifically by a terrestrial *S. albus* to provide it with a defence mechanism against bacteriophages.

There is little doubt that PVA 94-07 and GBA 94-10 are close relatives of *S. albus* J1074, as evident from the genomes' synteny and lower, compared to other *Streptomyces* analyzed, numbers of co-orthologs present in the former strains. Comparative genomics applied to the three strains helped to identify some candidate genes absent in *S. albus* J1074 but present in sponge isolates that might be responsible for partial marine adaptation of the latter. Since the adaptation phenotype is, however, quite subtle, it is not clear whether inactivation of identified potential MAG genes will lead to a clear conclusion. Transfer of the identified partial NDH-1 operon from one of the marine species to *S. albus* J1074 could be a better approach, if it enables the recombinant strain to grow and differentiate normally on the medium with sea water.

Interesting results were obtained after mining for secondary metabolite gene clusters in the three genomes and their comparative analysis. Biosynthesis of secondary metabolites was suggested to play a certain role in environmental adaptation [6]. From this point of view, acquisition of new, compared to *S. albus* J1074, biosynthetic gene clusters by marine isolates may be relevant for such an adaptation, reflecting different microbial communities the bacteria had been exposed to. Notably, most of the newly acquired clusters are associated with transposons, and at least one of them, specifying biosynthesis of enterocin, resembles a giant composite transposon-like element. This *enc* cluster can be used in future experiments designed to study mechanisms of cluster transfer between different species.

Localization of some secondary metabolite gene clusters or parts thereof as inverted copies within long TIRs on the linear chromosomes of the studied streptomycetes raises interesting questions regarding the recombination events behind. How did these inverted copies arise? Are they functional? Can one copy or part thereof be lost due to recombination between TIRs? Can unexpected recombination lead to fusion of one copy to another cluster? At least for *S. albus* J1074 this seems to be the case, as one hybrid gene cluster at the 5′ end of its chromosome seems to have been generated by such an event. Functional antibiotic biosynthesis gene cluster has been identified within TIR of a linear chromosome of *Streptomyces ambofaciens* [38]. Subsequent analysis of TIRs of the linear chromosomes of two *S. ambofaciens* strains indicated their extensive rearrangement during evolution via insertion or loss of DNA fragments, followed by homogenization via recombination [39]. Detailed studies of these phenomena may help to unravel the mechanisms by which secondary metabolite biosynthesis evolves in *Streptomyces* bacteria, and the ways to rationally design hybrid gene clusters for the production of new bioactive compounds.

## Supporting Information

File S1 **This supporting information file includes the following: Table S1.** Comparative analysis of conserved genes in *Streptomyces* sp. isolates MP94-07 and P94-10. **Table S2.** Specific genes absent in *S. albus* and terrestrial streptomycetes studied, but found in PVA 94-07, GBA 94-10 and marine actinobacteria genomes. **Table S3.** Genes from the MAG pool identified in the genomes of PVA 94-07, GBA 94-10 and *S. griseus*. **Figure S1.** 16S rRNA gene-based neighbor-joining phylogenetic tree of actinomycetes. isolated from *Geodia barretti* with bootstrap values (1000 replications). **Figure S2.** 16S rRNA gene-based neighbor-joining phylogenetic tree of actinomycetes isolated from *Phakellia ventilabrum* with bootstrap values (1000 replications). **Figure S3.** Genome synteny between the *S. albus* J1074, *Streptomyces* sp. PVA 94-07, and *Streptomyces* sp GBA 94-10.
(DOC)
