# Supplementary material for: Genomics of Sponge-Associated Streptomyces spp. Closely Related to Streptomyces albus J1074: Insights into Marine Adaptation and Secondary Metabolite Biosynthesis Potential
Source: PLoS One. 2014 May 12;9(5):e96719. doi: 10.1371/journal.pone.0096719 (PMC4018334; doi:10.1371/journal.pone.0096719)

**Supporting information**

Ian et al. Comparative genomics and genome mining of sponge-associated *Streptomyces* spp. closely related to *Streptomyces albus* J1074: insights into partial marine adaptation and secondary metabolite biosynthesis potential.

**Table S1.** Comparative analysis of conserved genes in *Streptomyces* sp. isolates MP94-07 and MP94-10.

| **Annotation** | **MP94-07 vs. MP94-10 identity, %** | | **Closest database match to the MP94-07 gene sequence** |
| --- | --- | --- | --- |
|  | **Gene** | **Protein** |  |
| DNA-directed RNA polymerase,  alpha sunbunit | 100 | 100 | 99.2 %, *Streptomyces albus* J1074 (NZ_ABYC01000223) |
| Glycerol kinase  *glpF* | 99.9 | 100 | 98.8 %, *Streptomyces albus* J1074 (NZ_ABYC01000092) |
| Guanylate kinase | 100 | 100 | 99.1 %, *Streptomyces albus* J1074 (NZ_DS999645) |
| Phenylalanyl-tRNA synthetase, alpha subunit | 99.7 | 100 | 98.8 %, *Streptomyces albus* J1074 (NZ_DS999645) |
| RecA recombinase | 100 | 100 | 98.7 %, *Streptomyces albus* J1074 (NZ_DS999645) |
| Ribosomal protein S2 | 100 | 100 | 99.1 %, *Streptomyces albus* J1074 (NZ_ABYC01000381) |
| Ribosomal protein S5 | 100 | 100 | 99.0 %, *Streptomyces albus* J1074 (NZ_ABYC01000223) |
| Ribosomal protein L5 | 100 | 100 | 99.6 %, *Streptomyces albus* J1074 (NZ_ABYC01000223) |
| Ribosomal protein L11 | 100 | 100 | 99.8 %, *Streptomyces albus* J1074 (NZ_ABYC01000225) |
| Triosephosphate isomerase | 99.6 | 99 | 98.3 %, *Streptomyces albus* J1074 (NZ_ABYC01000125) |

^a^2 nucleotide substitutions

^b^3 nucleotide substitutions

**Table S2.** Specific genes absent in *S. albus* and terrestrial streptomycetes studied, but found in PVA 94-07, GBA 94-10 and marine actinobacteria genomes.

| **PVA 94-07** | **GBA 94-10** | **Marine Actinobacteria** | **Marine *Streptomyces*** | **Annotation** |
| --- | --- | --- | --- | --- |
| B590_21402 | B591_21557 | *Rhodococcus erythropolis* PR4 DNA | *Streptomyces* sp. PP-C42 | hypothetical protein |
| B590_25389 | B591_25588 | *Janibacter* sp. HTCC2649 | *S*. sp. PP-C42; *S. xinghaiensis* S187 | hypothetical protein |
| B590_00869 | B591_00919 | *Janibacter* sp. HTCC2649 | *S*. sp. PP-C42; *S. sulphureus* L180 | peptidase S58 DmpA |
| B590_13288 | B591_13148 | *Salinispora arenicola* CNS-205 | *S*. sp. PP-C42 | amidohydrolase |
| B590_07610 | B591_07535 | Marine actinobacterium PHSC20C1 | *S*. sp. PP-C42 | metallophosphoesterase |
| B590_25409 | B591_25608 | *Sal. tropica* CNB-440; *Sal. arenicola* CNS-205 | *S. griseoaurantiacus* M045 | hypothetical protein |
| B590_13293 | B591_13153 | *Sal. arenicola* CNS-205 | *S*. sp. PP-C42 | NIPSNAP family protein |
| B590_13268 | B591_13128 | *Sal. arenicola* CNS-205 | *S*. sp. PP-C42 | glyoxalase |
| B590_13348 | B591_13208 | *Sal. arenicola* CNS-205 | *S*. sp. PP-C42 | hypothetical protein |
| B590_17194 | B591_17199 | *J*. sp. HTCC2649 | *S*. sp. PP-C42 | hypothetical protein |
| B590_16879 | B591_16884 | Sal. tropica CNB-440; Sa. arenicola CNS-205; Actinobacterium marinum DSM 15272; J. sp. HTCC2649; M. actinobacterium PHSC20C1 | *S*. sp. PP-C42; *S. xinghaiensis* S187 | TrkA-N domain-containing protein |
| B590_16884 | B591_16889 | Sal. tropica CNB-440; Sal. arenicola CNS-205; *A. marinum* DSM 15272; M. actinobacterium PHSC20C1 | *S*. sp. PP-C42; *S. xinghaiensis* S187 | TrkH family potassium uptake protein |
| B590_01039 | B591_01094 | *Sal. tropica* CNB-440; *Sal. arenicola* CNS-205; M. actinobacterium PHSC20C1 | *S*. sp. PP-C42; *S. sulphureus* L180 | TetR family transcriptional regulator |
| B590_21357 | B591_21512 | *R. erythropolis* PR4 DNA | S. sp. PP-C42 | hypothetical protein |
| B590_11855 | B591_11771 | *R. erythropolis* PR4 DNA; *A. marinum* DSM 15272 | *S. griseoaurantiacus* M045; *S*. sp. PP-C42 | ABC sugar transporter |
| B590_01059 | B591_01114 | *R. erythropolis* PR4 DNA | - | hypothetical protein |
| B590_13363 | B591_13223 | *Sal. arenicola* CNS-205 | - | FAD dependent oxidoreductase |

**Table S3.** Genes from the MAG pool identified in the genomes of PVA 94-07, GBA 94-10 and *S. griseus.*

| **PVA 94-07 (locus tags)** | **GBA 94-10 (locus tags)** | ***S. griseus subsp. griseus* NBRC 13350 (locus tags)** | **Gene symbol** |
| --- | --- | --- | --- |
| B590_00884 | B591_00934 | SGR_785 | *nuoN* |
| B590_00889 | B591_00939 | SGR_786 | *nuoM* |
| B590_00894 | B591_00944 | SGR_787 | *nuoL* |
| B590_00899 | B591_00949 | SGR_788 | *nuoK* |
| B590_00904 | B591_00954 | SGR_789 | *nuoJ* |
| B590_00909 | B591_00959 | SGR_790 | *nuoH* |
| B590_00919 | B591_00969 | SGR_792 | *nuoA* |

**Figure S1.** 16S rRNA gene-based neighbor-joining phylogenetic tree of actinomycetes. isolated from *Geodia barretti* with bootstrap values (1000 replications). The nearest neighbors revealed through BLAST search of non-redundant nucleotide sequences in the public databases are presented in the tree. Nucleotide sequence accession numbers are given in brackets. The scale bar corresponds to 0.02 substitutions per nucleotide positions.

**Figure S2.** 16S rRNA gene-based neighbor-joining phylogenetic tree of actinomycetes isolated from *Phakellia ventilabrum* with bootstrap values (1000 replications). The nearest neighbors revealed through BLAST search of non-redundant nucleotide sequences in the public databases are presented in the tree. Nucleotide sequence accession numbers are given in brackets. The scale bar corresponds to 0.02 substitutions per nucleotide positions.

**Figure S3.** Genome synteny between *Streptomyces* sp. PVA 94-10, and *Streptomyces* sp GBA 94-07. The diagram shows X/Y plots of dots forming syntenic regions between the genomes.


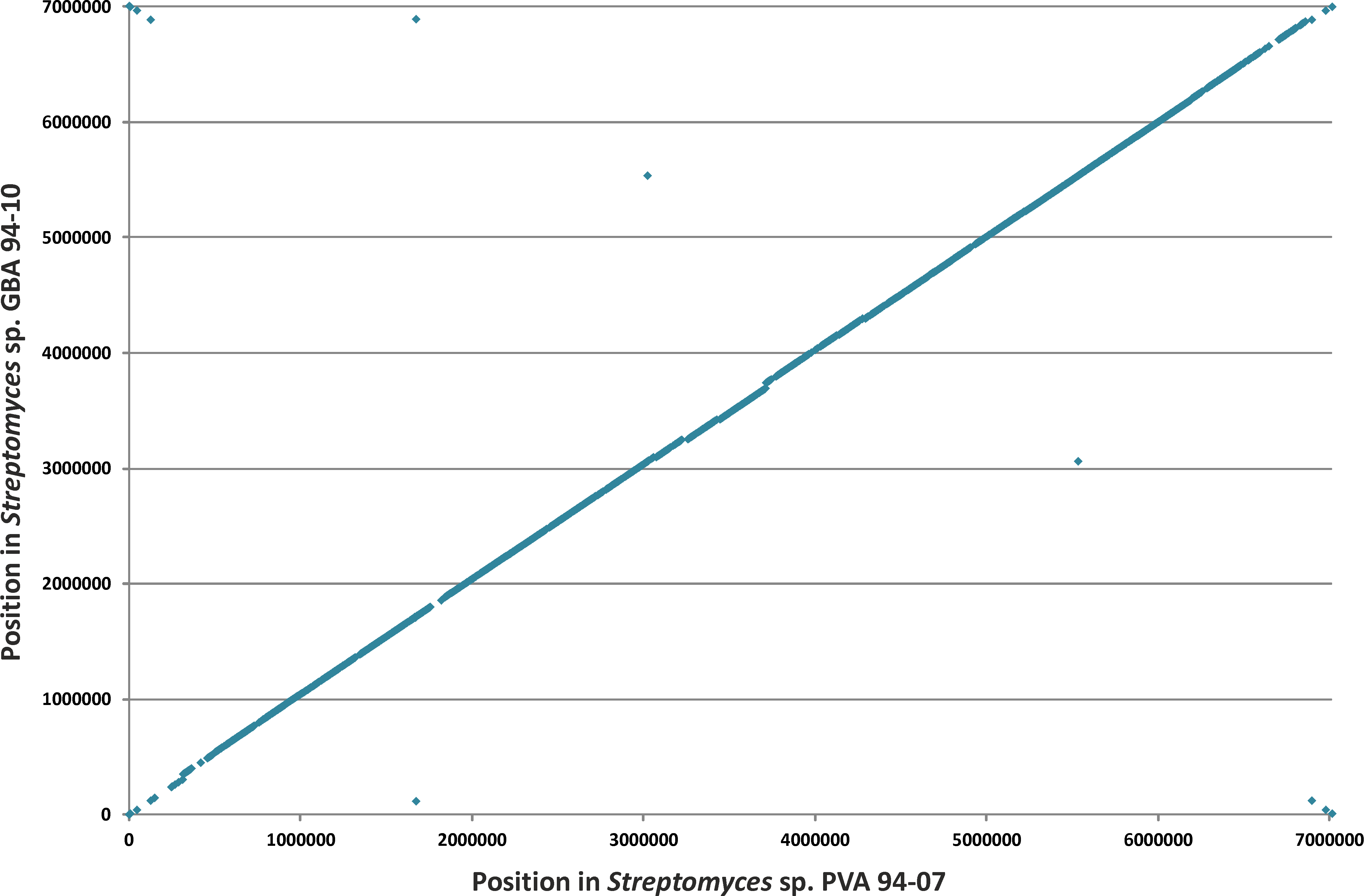


**Figure S4.** Genome synteny between the *Streptomyces* sp. GBA 94-10 and *S. albus* J1074. The diagram shows X/Y plots of dots forming syntenic regions between the genomes. The *S. albus* J1074 data were obtained from GenBank database (NC_020990).


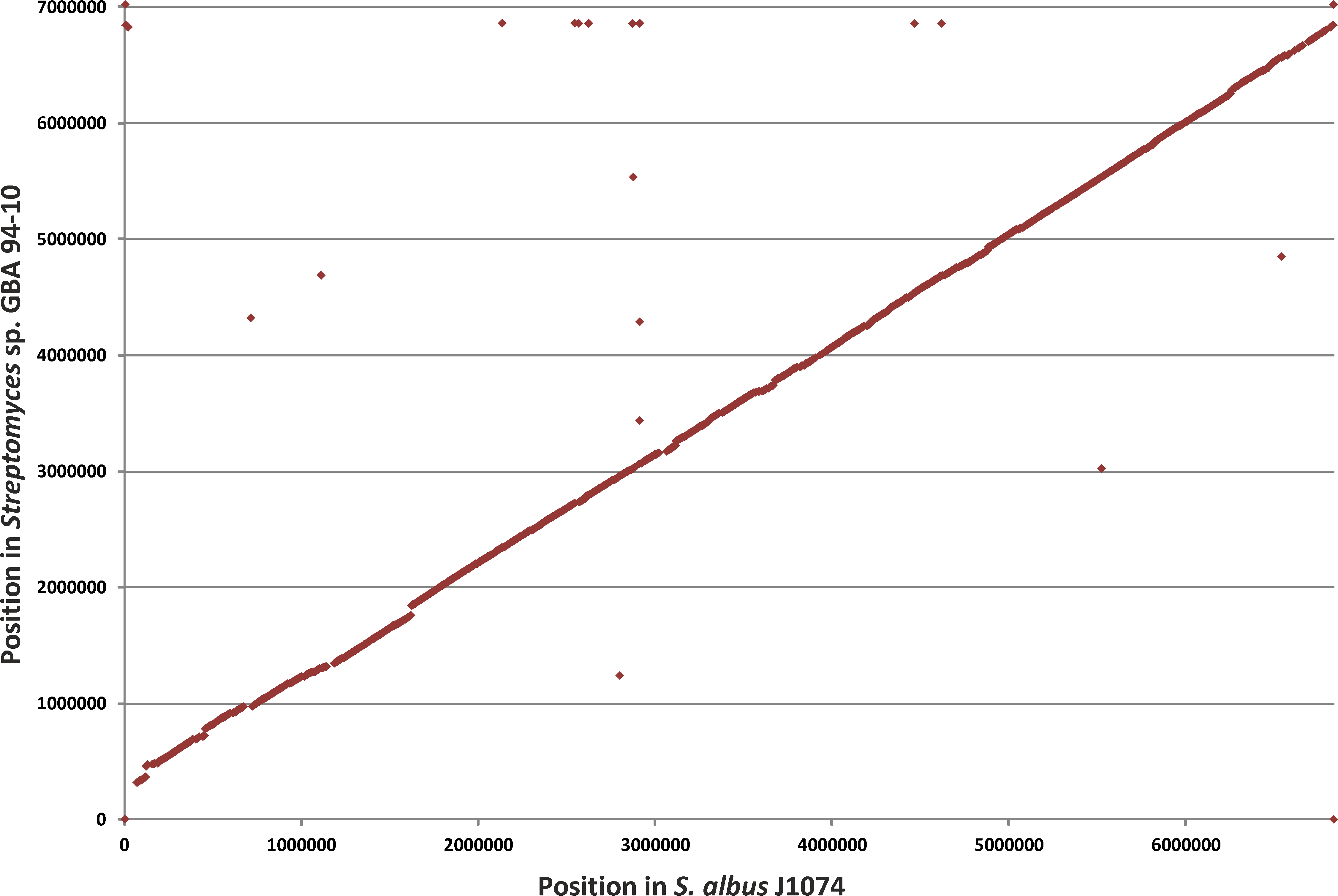


**Figure S5.** Genome synteny between the *Streptomyces* sp. PVA 94-07 and *S. albus* J1074. The diagram shows X/Y plots of dots forming syntenic regions between the genomes. The *S. albus* J1074 data were obtained from GenBank database (NC_020990).


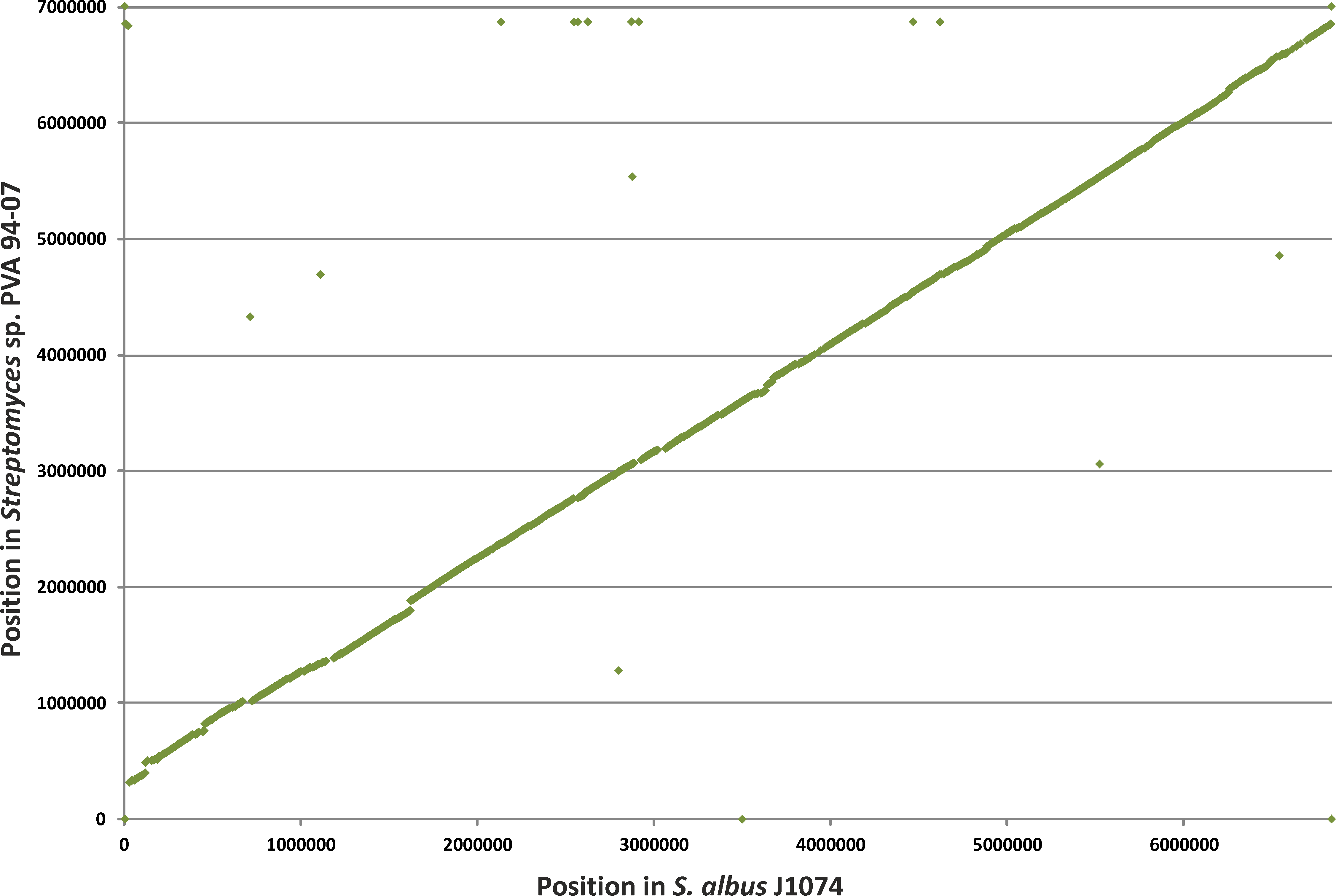

Supplement: File S1 — This supporting information file includes the following: Table S1. Comparative analysis of conserved genes in Streptomyces sp. isolates MP94-07 and P94-10. Table S2. Specific genes absent in S. albus and terrestrial streptomycetes studied, but found in PVA 94-07, GBA 94-10 and marine actinobacteria genomes. Table S3. Genes from the MAG pool identified in the genomes of PVA 94-07, GBA 94-10 and S. griseus. Figure S1. 16S rRNA gene-based neighbor-joining phylogenetic tree of actinomycetes. isolated from Geodia barretti with bootstrap values (1000 replications). Figure S2. 16S rRNA gene-based neighbor-joining phylogenetic tree of actinomycetes isolated from Phakellia ventilabrum with bootstrap values (1000 replications). Figure S3. Genome synteny between the S. albus J1074, Streptomyces sp. PVA 94-07, and Streptomyces sp GBA 94-10. (DOC) [file pone.0096719.s001.doc]
